# The effects of menstrual cycle phase on physical performance in female soccer players

**DOI:** 10.1371/journal.pone.0173951

**Published:** 2017-03-13

**Authors:** Ross Julian, Anne Hecksteden, Hugh H. K. Fullagar, Tim Meyer

**Affiliations:** 1 Institute of Sports and Preventive Medicine, Saarland University, Saarbruecken, Germany; 2 Sport & Exercise Discipline Group, University of Technology Sydney, Lindifield, Australia; 3 Department of Athletics (Football), University of Oregon, Leo Harris Pwky Drive, Eugene, Oregon, United States of America; Universidad Europea de Madrid, SPAIN

## Abstract

**Background:**

Female soccer has grown extensively in recent years, however differences in gender-specific physiology have rarely been considered. The female reproductive hormones which rise and fall throughout the menstrual cycle, are known to affect numerous cardiovascular, respiratory, thermoregulatory and metabolic parameters, which in turn, may have implications on exercise physiology and soccer performance. Therefore, the main aim of the present study was to investigate potential effects of menstrual cycle phase on performance in soccer specific tests.

**Methods:**

Nine sub elite female soccer players, all of whom have menstrual cycles of physiological length; performed a series of physical performance tests (Yo-Yo Intermittent endurance test (Yo-Yo IET), counter movement jump (CMJ) and 3x30 m sprints). These were conducted at distinct time points during two main phases of the menstrual cycle (early follicular phase (FP) and mid luteal phase (LP)) where hormones contrasted at their greatest magnitude.

**Results:**

Yo-Yo IET performance was considerably lower during the mid LP (2833±896 m) as compared to the early FP (3288±800 m). A trend towards significance was observed (p = 0.07) and the magnitude based inferences suggested probabilities of 0/61/39 for superiority/equality/inferiority of performance during the mid LP, leading to the inference of a possibly harmful effect. For CMJ (early FP, 20.0±3.9 cm; mid LP 29.6±3.0 cm, p = 0.33) and sprint (early FP, 4.7±0.1 s; mid LP, 4.7±0.1 s, p = 0.96) performances the results were unclear (8/24/68, 48/0/52, respectively).

**Conclusion:**

The results of this study are in support of a reduction in maximal endurance performance during the mid LP of the menstrual cycle. However, the same effect was not observed for jumping and sprint performance. Therefore, consideration of cycle phase when monitoring a player’s endurance capacity may be worthwhile.

## Introduction

The professionalism and interest in female soccer has rapidly increased over the last decade, which has led to an expediential rise in research within many realms of the game. Despite the increasing amount of scientific work surrounding female soccer, gender-specific aspects of physiology, particularly the menstrual cycle and its effects on the physical performance, has been unaccounted for and remains fundamentally unknown[1, 2]. The menstrual cycle encompasses two main phases, the follicular phase (FP) and the luteal phase (LP). The follicular phase can split further into two sub phases; the early FP, which is characterised with low concentrations of both the key hormones oestrogen and progesterone; and the mid FP where oestrogen is high independently from progesterone. The LP is typified by high concentrations of both oestrogen and progesterone. These two main phases are separated by a steep surge in luteinizing hormone triggering ovulation. These cyclical changes are said to be often predictable whilst spanning over the reproductive years[3].

Besides from reproductive function female sex hormones are further known to affect numerous cardiovascular, respiratory, thermoregulatory and metabolic parameters. Which may plausibly be expected to have implications on exercise physiology, for example via fluid retention, changes in body temperature, and energy metabolism[4]. Currently, much of the research has investigated these effects on endurance performance, and the results thus far have been highly equivocal[5–14]. The differing results of the studies to date, may be due to methodological differences. Inconsistent time points within the menstrual cycle when performance had been assessed, the method of assessment (varying performance tests), the verification of cycle phases and/or assessment of physiological cycles, could be factors for the varied findings.

Currently, to the authors knowledge only three studies have evaluated the effect of menstrual phase on intermittent activity and related performance[15–17]. Two of the three studies indicated no differences between cycle phase. However, Middleton and Wenger[15] found a significant enhancement in power output during ten, six second maximal sprints on a cycle ergometer in the LP. Although the authors found only small average differences in power output, the improvement during the LP was exhibited in every subject. Moreover, if these results were translated into running time, this difference in work would translate to approximately one-meter difference in a six second sprint, which in terms of soccer, could make the difference in reaching the ball at important stages in a match. However, throughout these studies, the population sample was primarily healthy active females and not those of a high competitive level and studies were not related to soccer.

The current study aims to determine whether menstrual cycle phase influences a series of soccer related physical performance parameters in a high levelled soccer specific population. Based on the above considerations, the designs involve a strict protocol for the assessment of physiological cycles and distinct timing of performed tests in relation to cycle phases.

## Materials and methods

### Subjects

**Fig 1:**
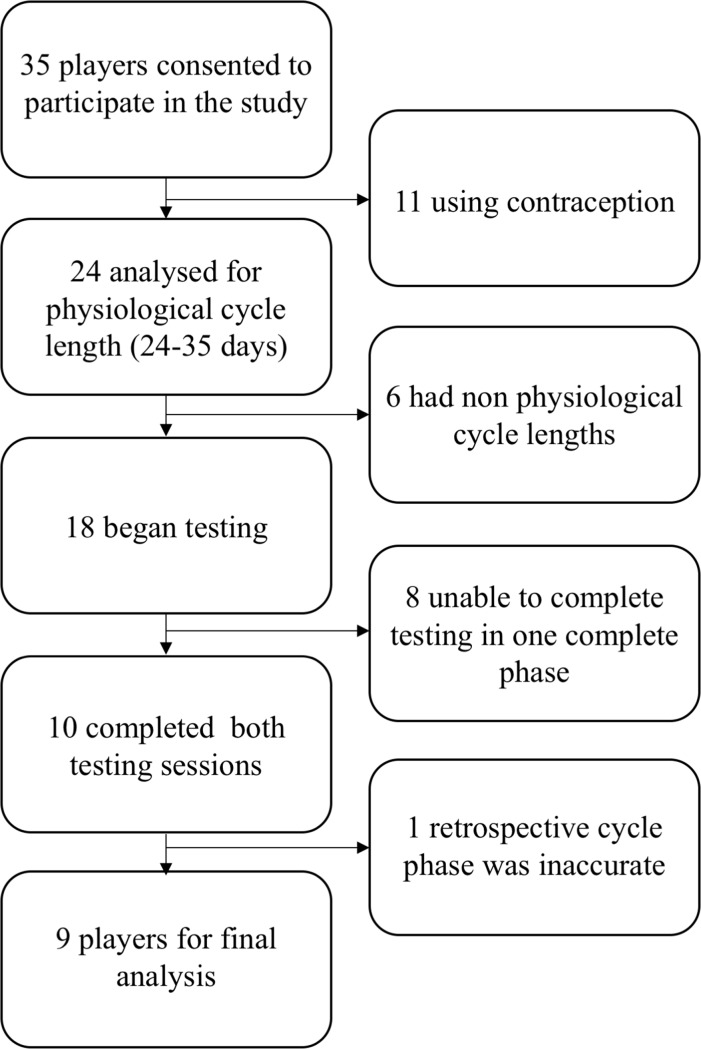
Details of the participants’ timeline throughout the study.

Thirty-five high-level female soccer players agreed to participate in the study. Formal sample size calculations were not performed, all players of the local second league female football team who fulfilled the inclusion criteria were enrolled for the study. The inclusion criteria for participation in the study were: 1) the absence from any form of contraception (oral, implanted, injected, intrauterine devices, patches), 2) regular cycle of physiological length (24–35 days[8]), 3) free from any illness or disease which could affect performance and/or health, 4) not suffering from an injury which would affect their performance, 5) participated in competitive soccer for a minimum of five years. A further inclusion criteria was that the testing must be completed over the 24–35 day cycle (reducing the influence of confounding variables including but not limited to: humidity, ambient temperature). Therefore, nine participants (age: 18.6 ± 3.8 y, height: 161.2 ± 6.6 cm, weight: early FP 59.1 ± 7.7 kg, mid LP 58.8 ± 7.5 kg) were suitable for analysis; the flow of the participants is represented in Fig 1. These players all competed in the second women’s league (2nd Frauen-Bundesliga), Germany. The players were fully informed of all experimental procedures before giving their written informed consent to participate. If players were younger than 18 years of age, parental written consent was obtained. The current study was conducted in accordance with the declaration of Helsinki and approved by the local ethics committee (Aerztekammer des Saarlandes, approval number 130/14).

### Study design

The observational design implemented in this study investigated the differences in physical performance parameters during two distinct phases of the menstrual cycle. The participants completed a battery of physical tests, measuring several components of soccer specific physical performance capacity. These included; lower limb power, sprinting capability and endurance capacity[18]. The participants did not conduct any vigorous exercise on the days of testing and the training the day before testing days remained consistent; training comprised of low/moderate aerobic training for 30–45 minutes followed by 30 minutes of strength training. The tests were conducted during two time points, representative of the early follicular and mid luteal phases, respectively (early FP days 5–7 and mid LP days 21–22[19]), where hormonal concentrations differ greatest between the two time points[19, 20]. Data was collected in the early evening (during their normal training session), and training time was kept consistent for the duration of the study. Testing sessions were conducted during the second half of the season for over an 8-week period (March—May). All measures were conducted outside on artificial turf under similar weather conditions. If weather was deemed unsuitable and at risk of influencing results, and or to players’ welfare, (e. g. any form of rain) the test was either cancelled or if possible re-scheduled.

### Determination of menstrual phase

**Fig 2:**
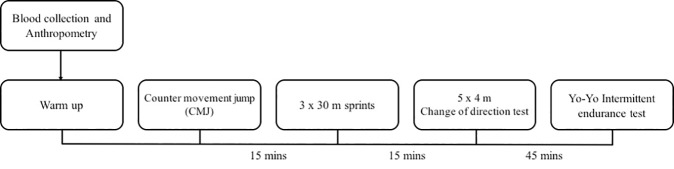
Timeline of the testing protocol.

Twice a month participants completed a menstruation diary, which included: date of menses, length of menses, and severity of blood flow and discomfort. This information was recorded for a minimum of 6 months, for a valid characterisation of an individual’s menstrual cycle[19]. From retrospective analysis, (counting back from previous menses, from month to month, indicating the length of the cycle) the next cycle phase was then prospectively determined[19] and time-point was calculated. For final confirmation of LP sampling time, the participants completed menstruation diaries during the course of testing. Consequently, one player dropped out due to LP sampling time being incorrect (Fig 2). Furthermore, serum oestrogen and progesterone were used to verify physiological reproductive function and verify timing of tests with respect to the cycle phases[19].

### Outcome measures

The following panel of established tests for components of soccer specific physical performance capacities were applied and conducted in this order (Fig 2): 1) Jump height during a counter movement jump (five trials, mean of the best three) was implemented as an indicator of lower-limb power[21], 2) 30 m (recording set at 5, 10 and 30 m) sprint time was employed as a parameters of sprint capability (fastest of three trials)[22], 3) running distance in the Yo-Yo IET was used as a measure of football-specific endurance capacity[23].

#### Counter movement jump (CMJ)

Characteristics of the CMJ were determined using a force platform (Quattro Jump, Type 9290AD, Kistler Instrument AG, Winterthur, Switzerland) and analysed using professional motion analysis software (Contemplas Bewegungsanalyse, Contemplas Gmbh, Kempten, Germany). Jump height was determined as the centre of mass displacement, calculated from the recorded force and body mass. The CMJ began from an upright position, making a downward movement to a knee angle of approximately 90° and simultaneously beginning to push-off, whilst hands are placed upon their hips, with a rest period of 30 s between efforts.

#### 3x30m sprints

Players were asked to stand with their toe behind a white line (touchline of the soccer pitch), and ran maximally straight ahead for 30m. 30 m sprint recording times at 5 m, 10 m and 30 m was employed and measured by light gates and a hand-held monitor (Brower Timing System, Utah, USA). Once a sprint was completed the players had 2 mins of recovery and then repeated the test following the same procedure for all three trials.

#### Yo-Yo Intermittent endurance test (Yo-Yo IET)

The test was performed outdoors on artificial turf similarly described by Bangsbo et al[24]. Concisely, the test consisted of repeated 2 x 20 m runs with an 180° turn in between at a progressively increasing speed controlled by audio beeps. Between each running bout, the players had a 5 s rest period, during the active break in the form of a 2.5 m walk. Termination of the test occurred when a player had twice failed to reach the finish line, in time. Total distance of completed shuttles was recorded as the test result. All players had been familiarised with the test procedures previously. HR was measured before the warm-up period (rest) and was continuously measured using commercial heart rate monitors (Polar, Kempele, Finland) until exercise cessation (HR_peak_). Blood lactate (BLa) was collected in 25μl samples of capillary blood, withdrawn from the earlobe and were subsequently measured using an enzymatic method (Super GL, Rolf Grenier, Biochemica, Flacht, Germany). BLa was collected prior to exercise (rest value), then further collected immediately after, 1 min, 3 min and 5 min post-test. HR (≥ 220 –age (years)), BLa concentration (≥ 8 mmol/l) and RPE were used as an objective criteria of exhaustion[25].

#### Blood collection

Venous blood samples were collected on the day of testing prior to any form of activity. These samples were obtained from the anti-cubital vein by a standard protocol, following 10–15 min of seated rest. The participants did not perform any vigorous exercise on the day of blood sampling. Blood samples were transported immediately to the laboratory for appropriate procedures. Serum tubes were centrifuged at 4000 revolutions per min for 5 min, aliquoted and then stored frozen at -80°C within 2 h of sampling. Hormones were measured in an accredited medical laboratory. Oestrogen was measured using an Estradiol II electrochemiluminescence immunoassay ECLIA (IBL International GMBH, Hamburg, Germany) with intra- and inter-assay coefficients of 4.13–6.81% and 6.72–9.39%, respectively. Progesterone was measured using a 17-OH-Progesterone enzyme immunoassay ELISA (IBL International GMBH, Hamburg, Germany) with intra- and inter-assay coefficients of 2.8–4.9% and 5.8–9.2%, respectively.

### Statistics

Data analysis was conducted using the statistical software package SPSS v.21 (SPSS Inc., Chicago, IL, USA). All data were normally distributed (Kolmogorov-Smirnov) and expressed as means ± standard deviations (SD). Students paired *t*-tests were conducted to determine the difference in mean response between phases for all variables. The magnitude of the mean differences between phases were expressed as effect sizes (ES) [26]. The extent of the ES are classified as follows: trivial (<0.2), small (>0.2–0.6), moderate (>0.6–1.2), large (>1.2–2.0), very large (>2.0), based on updated guidelines from Batterham and Hopkins[27]. Magnitude-based inferences (MBI) were conducted to determine the possible benefit, in terms of beneficial or harmful effects, of the cycle phase. This approach represents a contemporary method of data analysis that uses confidence intervals to calculate the probability that a difference is practically meaningful. For assessing the difference in team performance smallest worthwhile change was set at Cohen effect size of 0.2[27]. When clear interpretation was possible, a qualitative descriptor was assigned to the following quantitative chances of performance effect: 0.5% to 5%, very unlikely; 5% to 25%, unlikely; 25% to 75%, possibly; 75% to 95%, likely; 95% to 99.5%, very likely; and >99.5%, most likely[27].

## Results

The participants body mass and sum of four skin folds were not significantly different between menstrual phases (Table 1). Oestrogen and progesterone levels of the players were significantly higher in the mid LP compared to the early FP.

**Table 1:** Anthropometric and hormone characteristics of females involved in the study. Comparison between Follicular and Luteal phase.

Variable		Follicular Phase	Luteal Phase
	Means ± SD	Means ± SD	Means ± SD
Age (y)	19 ± 4		
Height (cm)	161.3 ± 6.6		
Body mass (kg)		59.1 ± 7.7	58.8 ± 7.5
Body fat (%)		17.7 ± 2.5	17.8 ± 2.2
Oestrogen (pg/ml)		26.8 ± 22.1	109.8 ± 55.6*
Progesterone (nmol/l)		2.2 ± 0.6	6.5 ± 2.0*

*Note*: Body fat calculated by the sum of four skinfolds (biceps, triceps, subscapular, suprailiac).

* indicates a significant difference from early FP to LP P < 0.05.

**Fig 3:**
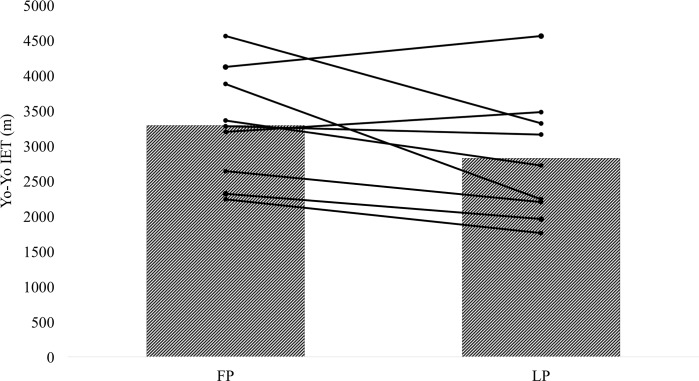
Mean meters of completion during the Yo-Yo IET during the two phases of the menstrual cycle. The data for each individual has been superimposed onto each group mean. The menstrual phases are abbreviated as FP (early follicular phase) and LP (mid luteal phase).

In Yo-Yo IET performance, MBI intimated the qualitative inference that there was a possibly harmful effect for maximal performance in the LP, whereas, all other performance variables were found to be unclear. Moreover, the ES for the Yo-Yo IET was small to moderate, whereas all other variables were trivial (Table 2). For all performance variables, there were no significant differences revealed, although a trend towards significance was observed in the Yo-Yo IET between phases (p = 0.07) (Fig 3).

**Table 2:** Performance outcomes for all parameters measured during the follicular phase and luteal phase. Results expressed as means ± standard deviations, statistical significance, effect sizes and magnitude based inferences.

Variables	FP	LP	*P*	ES	% Chance +/trivial/-	Qualitative Inference
0-5m (s)	1.1 ± 0.1	1.1 ± 0.1	1.00	0.00	50/0/50	Unclear
0-10m (s)	1.9 ± 0.1	1.9 ± 0.1	0.25	0.18	10/7/83	Unclear
0-30m (s)	4.7 ± 0.1	4.7 ± 0.1	0.96	0.00	48/0/52	Unclear
CMJ (cm)	29.0 ± 3.9	29.6 ± 3.0	0.33	0.16	8/24/68	Unclear
Yo-Yo IET (m)	3289 ± 801	2822 ± 896	0.07	0.56	0/61/39	Possibly harmful

Note: FP = Early follicular Phase; LP = Mid luteal Phase; ES = Effects Size; MBI = Magnitude Based Inference.

There was a likely beneficial inference for HR pre values tending towards the early FP, whereas the HR post values were deemed unclear. RPE was also deemed unclear. Lactate values were inferred to be unclear pre-and 1 min post the Yo-Yo test. However, at 3 and 5 min post, the MBIs suggested that it was very likely harmful during the early FP, where lactate values were higher. The effect sizes for all variables ranged from trivial to moderate (Table 3). There were significant differences between phases for HR prior to exercise and lactate values at minutes 1 and 3 post Yo-Yo test (p = 0.04, p = 0.01 and p = 0.03, respectively). For HR post, lactate pre-and lactate 1 min post Yo-Yo test and RPE there were no significant differences observed (p > 0.05).

**Table 3:** Internal load measures during the follicular phase and luteal phase. Results expressed as means ± standard deviations, statistical significance, effect sizes and magnitude based inferences.

Variables	FP	LP	*P*	ES	% Chance +/trivial/-	Qualitative Inference
HR Pre (bpm)	97 ± 16	105 ± 12	0.04	0.45	91/9/0	Likely beneficial
HR post (bpm)	194 ± 4	193 ± 5	0.90	0.05	27/37/36	Unclear
RPE (AU)	18.7 ± 0.7	18.6 ± 0.9	0.76	0.14	23/31/46	Unclear
Lactate pre (mmol L^-1^)	2.3 ± 0.8	2.1 ± 0.8	0.65	0.21	17/25/58	Unclear
Lactate 1min post (mmol L^-1^)	9.3 ± 1.4	8.6 ± 0.7	0.20	0.41	6/28/66	Unclear
Lactate 3min post (mmol L^-1^)	8.8 ± 2.2	7.6 ± 1.6	0.01	0.75	0/1/99	Very likely harmful
Lactate 5min post (mmol L^-1^)	8.7 ± 2.2	6.9 ± 1.9	0.03	0.93	1/4/95	Very likely harmful

Note: FP = Early follicular Phase; LP = Mid luteal Phase; ES = Effects Size; MBI = Magnitude Based Inference; HR = Heart rate; RPE = Record of perceived exertion.

## Discussion

The purpose of the study was to investigate whether menstrual cycle phase influences different facets of physical performance in a group of high level soccer players. The main finding of the study suggested that Yo-Yo IET performance was affected during the LP of the menstrual cycle. However, in all other aspect of measured performance were not different between phases.

To the authors knowledge this is the first study to investigate the effects of menstrual cycle phase on different sport specific physical parameters in female soccer players. In the nine players, menstrual cycle phase appeared to have an effect on maximal endurance capabilities, as demonstrated through the Yo-Yo IET. It has been suggested that physical performance in elite female soccer is highly related to their trained status and maximal capacities[28], therefore, it may be postulated that the maintenance of these high levels throughout the entire cycle is important for success. In 78% of our population, a reduction of meters completed was observed in the mid LP (Fig 3). The results of the current study are in line with the extensive work of Lebrun et al[8]. Lebrun and colleagues used a cohort of 16 trained athletes (age 27.6 ± 3.8 y, height 167.9 ± 5.3 cm, mass 59.6 ± 6.7 kg, VO_2max_ 53.7 ± 0.9 ml^.^kg^-1.^min^-1^), who participated in a variety of sports including running, cycling, triathlon, squash, cross-country skiing, ultimate frisbee and rowing. This study contained a strict phase confirmation comparable to our trial. Through the use of a progressive and continuous treadmill running test, the results indicated that maximal endurance capacity was reduced in the LP.

Differences in endurance performance during various phases of the menstrual cycle have been postulated to be due to differences in heat regulation, substrate availability, and metabolism[6]. It is well documented that a female’s basal body temperature can differ between phases in the region of 0.3–0.5°C[19]. This elevation in temperature has been attributed to the surge of progesterone exhibited during the LP. The associated increase in body temperature has been suggested to limit prolonged exercise capabilities and increase cardiovascular strain[19]. This has been seen in previous studies, presenting higher VO_2_ values, accompanied with higher heart rates and RPE values during exercise at given percentages of VO_2max_[29–31]_._ Although basal body temperature was not measured in the present study, cardiovascular strain may have been present in our study and thus could influence performance. Conversely, it has been shown in previous studies, that exercise time to exhaustion is improved during the LP. This has been speculated that the role oestrogen plays on enhanced lipid metabolism, with spared glycogen and usually a supressed lactate response to exercise in the LP[32]. As seen in the present study, significant differences were found in lactate concentration between phases at 3 and 5 min post exercise. Therefore, there is the potential that the energy pathways may have been taxed differently between phase, for example it has been implicated previously that during the LP there is a lower contribution of anaerobic glycolysis and hence reduced La values[32]. A conclusive statement about this, however, is beyond the current scope of this study and perhaps requires deeper investigation with more sophisticated methodology (e. g. indirect calorimetry). Moreover, the present data, however, did not indicate any differences in lactate production at rest or at maximal lactate concentration, which was similarly found in female rowers completing a one-hour row at 70% VO_2max_[33].

It is important to consider the specific type of sports to validly assess if and how menstrual cycle phase potentially affects physical performance. However, in soccer (which is intermittent in nature), there is a special challenge to evaluate sport-specific physical performance, this is due to the current difficulty to establish valid and reliable tests sensitive to the sport, unlike in endurance sports. The Yo-Yo IET has been suggested to replicate physical patterns in female soccer[34] well, however, factors such as motivation have been suggested to cause differing results when being assessed at multiple time points, however, the exhaustion criteria was achieved in all players. In the present study, a reduction during the LP was observed in the clear majority of the players, with similar maximal HR, La and RPE values. Therefore, it can be postulated that although maximal effort was elicited in both phases, there was an observed reduction in maximal performance during the LP.

Sprinting performance in the present study did not differ between cycle phases at either split or full 30 m distance (Table 2). The absence of the menstrual cycle affecting sprinting performance in the present study concurs with previously published literature[35]. It had, however, been suggested previously that sprinting performance may differ between phases. An improvement in sprinting performance being attributed to the increase in basal body temperature during the LP[36]. However, a study from Somboonwong and colleagues[36] suggested that, although basal body temperature was significantly greater in the LP, sprinting performance over 40-yd (36.6m) was not affected. Unlike the present study, Somboonwong et al.,[36] only measured 40-yd sprinting time and did not investigate differing split times or acceleration. From the present study’s results, it could be postulated that cycle phase does not influence sprinting ability at many various aspects of acceleration and velocities. This was seen as 5 m, 10 m and 30 m sprint time was not affected by cycle phase. In soccer, a players’ ability to perform multiple high intensity actions and sprinting ability has been considered to be more important for overall performance than endurance capacity [37], therefore, the understanding that menstrual cycle phase does not affect sprinting behaviour is important for players and practitioners. This being said, in the present study and in the work from Somboonwong et al., sprinting performance was measured from singular sprints and not repeated sprint ability (RSA). RSA is another facet of soccer which has been suggested to be important for soccer performance. The only study to assess a repeated sprint and the effects of menstrual cycle phase, was conducted by Tsampoukos et al (2010). In the aforementioned study, eight females conducted two 30 s sprints with a 2 min passive recovery in between; the results indicated no effect of menstrual cycle phase on repeated sprint performance. However, this study, only employed sport science participants, not those of a sport specific population and, furthermore, only completed two sprints. Therefore, for future research, due to the lack of understanding and studies conducted in soccer, the investigation of menstrual cycle phase on RSA is warranted.

The menstrual cycle has been suggested to alter motor control and muscular strength. However, similarly to the present study de Jonge et al.,[38] found no differences between phases in 19 normally menstruating females for any strength parameter, including maximal isometric quadriceps strength with superimposed electrical stimulation, isokinetic knee flexion, and handgrip strength. It has been previously proposed that oestrogen, may have a strengthening action on skeletal muscle[39], although the mechanisms of this effect has not become clear. During the present study, the time points of measurements, were at minimal oestrogen (early FP) and when both progesterone and oestrogen were elevated (mid LP). Therefore, it can be speculated that the levels of oestrogen were not elevated enough to cause such an effect during early FP. Moreover, the antagonistic effect of progesterone, may have restricted the effects of oestrogen on muscular strength during LP[40]. Altogether, the current results suggest that CMJ was not effected by cycle phase, however, due to the assumptions of the effect of oestrogen on strength, future investigations should target other time points within the menstrual cycle, where oestrogen is independently elevated, i.e. in the rise prior to ovulation.

A limitation to the current study is that it did not deploy a randomised counter balanced design, whereby all participants first completed the testing in the early FP followed by the mid LP, this was due to the current method of cycle phase determination. Although the participants were well accustomed to this form of testing, confounding factors that may influence the current findings need to be considered. As recommended in previous literature, solely conducting menstruation calendars does not provide all relevant information regarding menstrual cycle phase[41]. Therefore, hormone values of oestrogen and progesterone were measured. However, concentrations were found to be lower than those previously described in the general population. In regards to research a progesterone limit has been recommended conservatively at 16 nmol/L[19]. It must be noted, that unfortunately at present reference values for athletic populations have yet to be defined and are not available. Furthermore, although the levels were not at the expected magnitude, changes in hormone values were observed; thus, the associated change in phase could be verified (Table 2). Moreover, the current data have been taken from the early FP and the mid LP; the associated hormones also differ just before ovulation in the late LP, where oestrogen is elevated without a pronounced presence of progesterone. Therefore, the independent effects of oestrogen have not been assessed where these results could also differ. Finally, although the subject number is in the range of previous studies[15–17], the results may not accurately represent a wider female soccer population.

## Conclusion

The current study indicates that there is potentially a reduction in maximal endurance performance during the LP of the menstrual cycle. However, this reduction in performance was not observed for jumping and sprint performance. Therefore, due to the findings of the current study, practitioners should keep menstrual cycle phase constant when completing routine physical assessments with their players, to ensure that changes in performance are consistent with the outcome and not due to the effects of the menstrual cycle. Alternatively, the cycle phase should at least be recorded and taken into account when interpreting the results.

## Supporting information

S1 Data Set Full data set including: Anthropometrics, football specific physical performance and hormonal concentration values. (PDF)
