# Supplementary material for: The effects of menstrual cycle phase on physical performance in female soccer players
Source: PLoS One. 2017 Mar 13;12(3):e0173951. doi: 10.1371/journal.pone.0173951 (PMC5348024; doi:10.1371/journal.pone.0173951)
Supplement: S1 Data Set — (PDF) [file pone.0173951.s001.pdf]

Subject number	Age (y)	Height (cm)	Weight (kg) FP	Weight (kg) LP	Body fat (%) FP	Body fat (%) LP
1	17	160	56.3	56.4	15.6	16.1
2	17	169	65.1	65.3	18.6	19.2
3	17	165	62.9	61	16.4	17
4	17	171.5	74.1	73.7	22.6	22
5	21	156.5	50.9	50.7	14.3	14.5
6	28	163.5	62.6	61.8	18.9	18.6
7	16	160	51.9	52.2	17.7	17.9
8	17	153.5	56.5	57	19.4	18.8
9	17	152.5	51.5	51.4	15.6	16

Note: FP = Follicular phase; LP = Luteal phase

Subject number	Phase	Counter movement jump (cm)	Sprint 5 m split (s)	Sprint 10 m split (s)	Sprint full 30 m (s)	Yo-Yo IET	Lactate pre	Lactate 1 min post	Lactate 3 min post	Lactate 5 min post	Heart rate pre	Max heart rate	RPE
1	Follicular	25	1.01	1.89	4.77	2320	2.72	9.75	8.97	8.83	110	195	19
2	Follicular	29	1.14	1.94	4.72	2640	2.1	8.18	7.66	7.45	106	193	18
3	Follicular	29	1.13	1.92	4.81	3360	1.85	9.50	8.84	8.80	90	186	19
4	Follicular	34	0.98	1.74	4.43	2240	4.2	12.4	11.6	11.4	115	196	20
5	Follicular	30	1.03	1.79	4.58	3880	2.19	9.25	9.60	9.59	74	195	18
6	Follicular	27	1.13	1.93	4.72	3200	1.82	8.06	7.41	6.82	82	189	18
7	Follicular	36	1.08	1.83	4.62	3280	1.6	9.84	9.16	9.03	120	196	19
8	Follicular	26	1.12	1.91	4.7	4560	2.27	8.51	8.73	8.71	85	195	18
9	Follicular	25	1.05	1.83	4.67	4120	1.8	7.8	7.59	7.35	89	198	19
1	Luteal	27	1.04	1.90	4.77	1960	2.03	8.10	7.87	7.59	114	195	19
2	Luteal	28	1.14	1.95	4.76	2200	2.33	8.53	6.15	5.8	106	198	20
3	Luteal	29	1.13	1.91	4.71	2720	3.79	8.11	7.81	3.52	92	191	19
4	Luteal	34	0.98	1.79	4.51	1760	1.99	9.64	9.18	9.24	117	199	19
5	Luteal	32	1.04	1.83	4.61	2240	1.82	9.45	8.92	8.51	102	185	17
6	Luteal	27	1.11	1.89	4.67	3480	2.1	8.79	7.26	5.18	88	189	19
7	Luteal	34	1.05	1.84	4.52	3160	2.24	7.55	7.01	7.15	123	196	18
8	Luteal	27	1.14	1.94	4.76	3320	1.67	8.89	7.86	8.84	96	196	18
9	Luteal	28	1.04	1.83	4.72	4560	0.94	8.50	7.08	6.23	106	192	18

Subject number	Oestrogen (pg/ml) FP	Oestrogen (pg/ml) LP	Progesterone (nmol/l) FP	Progesterone (nmol/l) LP
1	28.95	37.67	1.91	4.10
2	64.22	102.40	2.39	3.75
3	19.87	55.16	1.75	6.11
4	61.79	150.60	2.35	8.97
5	22.79	72.12	1.84	8.17
6	5.00	106.40	2.67	5.28
7	5.00	85.00	1.46	5.50
8	22.53	195.00	3.50	9.32
9	10.70	184.20	2.32	7.12

Note: FP = Follicular phase; LP = Luteal phase
